# Editorial: Diet and multiple sclerosis

**DOI:** 10.3389/fneur.2023.1347478

**Published:** 2023-12-12

**Authors:** Tyler J. Titcomb, Barbara S. Giesser, Scott M. Plafker, Ilana B. Katz Sand, Terry L. Wahls

**Affiliations:** ^1^Department of Internal Medicine, University of Iowa, Iowa City, IA, United States; ^2^Department of Epidemiology, University of Iowa, Iowa City, IA, United States; ^3^Pacific Neuroscience Institute, Santa Monica, CA, United States; ^4^Aging and Metabolism Research Program, Oklahoma Medical Research Foundation, Oklahoma City, OK, United States; ^5^Department of Cell Biology, University of Oklahoma Health Sciences Center, Oklahoma City, OK, United States; ^6^Department of Neurology, Corinne Goldsmith Dickinson Center for Multiple Sclerosis, Icahn School of Medicine at Mount Sinai, New York, NY, United States; ^7^Department of Neurology, University of Iowa, Iowa City, IA, United States

**Keywords:** multiple sclerosis, diet, registered dietitian nutritionists, experimental autoimmune encephalomyelitis, nutrition

## Introduction

Multiple sclerosis (MS) is a complex, immune-mediated neurodegenerative disease that affects an estimated 2.8 million people worldwide. MS onset is believed to result from interactions between genetic, epigenetic, and environmental factors. Over the past few decades, there has been a notable increase in the incidence of MS, suggesting significant changes in environmental risk factors associated with the development of the disease. In those with established MS, variability in prognoses not explained by genetics or differential disease-modifying therapies further highlights the importance of environmental factors as drivers of disease severity. This special edition called “*Diet and multiple sclerosis*” sheds light on the role of diet and nutrition in MS pathogenesis, management, and outcomes.

The article by Moles et al. investigates the gut microbiota, a fundamental mechanism by which diet likely impacts MS disease trajectory. Recent research has emphasized the gut-brain axis and its influence on neurological diseases. This study reveals that a specific phylum of bacteria, *Proteobacteria*, is more abundant in individuals with MS (*n* = 20) compared to healthy controls (*n* = 20). This study further found that individuals with MS have reduced production of short-chain fatty acids (SCFAs), particularly butyrate, which possesses known anti-inflammatory properties. Notably, both *Proteobacteria* abundance and low SCFA excretion correlated with worse MS prognosis. The article by Zyla-Jackson et al. further delves into mechanistic insights for the potential of dietary interventions in ameliorating the visual and motor deficits seen in experimental autoimmune encephalomyelitis (EAE), an animal model of autoimmune-mediated demyelination similar to MS. A ketogenic diet enriched with fiber preserved myelination, reduced inflammation, and enhanced overall neurological function by reducing key cytokines involved in mediating the infiltration, activation, and differentiation of auto-reactive T cells and neutrophils into the CNS.

Guglielmetti et al. evaluated the important question of which dietary components are linked to MS outcomes. This single center cross-sectional study conducted in Italy (*n* = 106), observed an association between ultra-processed food consumption and greater MS severity after accounting for other important clinical, demographic, and lifestyle covariates. While preliminary, these findings underscore the importance of exploring how diet quality can influence the course of MS. Corroborating these findings is the article by Goldin et al., which offers insights into the health behaviors of people with MS (*n* = 399) in Germany. This single center cross-sectional study observed that higher overall diet quality is associated with better performance on the nine-hole peg-test while accounting for age, sex, disease duration, and education. It is important to note that cross-sectional studies provide a snapshot of a population at a specific point in time, limiting their ability to establish causal relationships. People with MS who are more disabled may simply rely on convenient food options which tend to be ultra-processed and lead to poor diet quality. Additional prospective evidence, such as that from the article by Yu et al., is needed to fully elucidate the relationship between diet quality and MS outcomes. This secondary analysis of the Health Outcomes and Lifestyle in a Sample of people with MS (HOLISM) international longitudinal observational study at 7.5 years follow-up found that long-term ongoing adherence to the plant-based whole-food “Overcoming MS” (OMS) diet was associated with reduced risk of depression, severe fatigue, and severe disability among 671 people with MS while accounting for important clinical, demographic, and comorbidity covariates. Furthermore, a general healthy diet was also associated with reduced risk of depression. These findings support that long-term diet modifications could be a valuable lifestyle modification for symptom management among people with MS.

Data from clinical trials is ultimately needed to determine the relationship between diet and MS outcomes. The article by Wingo et al. investigated a unique approach to dietary management, time-restricted eating (TRE). This single-arm open-label pilot trial (*n* = 12) suggests that TRE, focusing on when, rather than what, to eat, is a feasible and acceptable intervention among people with RRMS. In the article by Villa et al. the impact of dietary changes on metabolic health and the influence on fatigue in RRMS is examined in a randomized parallel-arm trial (*n* = 77). Both the low-saturated fat and modified Paleolithic elimination diets led to within-group improvements in metabolic parameters and a reduction in fatigue, although the improvements in metabolic parameters did not mediate the effect on fatigue. This study considered several important demographic and clinical covariates and highlights the complex interplay between dietary interventions, metabolic health, and symptom management in MS. The results from these two trials indicate that dietary interventions may improve symptoms and warrant further investigation through randomized controlled trials.

The article by Titcomb et al. raises a critical issue: the need for nutritional guidance in the clinical care of people with MS. Given the strong desire among people with MS for evidence-based dietary guidelines, the inclusion of Registered Dietitian Nutritionists (RDNs) in the multidisciplinary care team for MS is proposed. The role of RDNs on the multidisciplinary care team can include assisting patients avoid the pitfalls of online dietary advice, promoting food literacy and provide support, screening for food insecurity and malnutrition, and preventing and managing comorbidities which are known to be associated with poorer prognosis among people with MS. Furthermore, the role of RDNs in MS care may extend beyond nutritional support to encompass counseling on food:drug interactions, research into long-term dietary impacts on MS outcomes, and education of other healthcare providers on the importance of nutrition in managing chronic diseases and preventing comorbidities.

The articles included in this special edition offer fresh insights into the diet-MS connection, and further support the promise of dietary modifications to alleviate symptoms and enhance quality of life. The growing recognition of nutrition's role in MS is evident from this research, paving the way for more effective and comprehensive strategies to manage MS-related symptoms. Collaborative efforts among researchers and clinicians (including RDNs) are central to fully realize the potential for nutrition in preventing and better managing MS-related symptoms and comorbid conditions linked to worse MS outcomes such as diabetes, hypertension, hyperlipidemia, and obesity. While further research is needed, these findings support the inclusion of RDNs on the MS multidisciplinary care team.

## Author contributions

TT: Writing—original draft, Writing—review & editing. BG: Writing—review & editing. SP: Writing—review & editing. IK: Writing—review & editing. TW: Writing—review & editing.

